# Identifying dysregulated pathways in cancers from pathway interaction networks

**DOI:** 10.1186/1471-2105-13-126

**Published:** 2012-06-07

**Authors:** Ke-Qin Liu, Zhi-Ping Liu, Jin-Kao Hao, Luonan Chen, Xing-Ming Zhao

**Affiliations:** 1 Institute of Systems Biology, Shanghai University, Shanghai, 200444, China; 2 School of Communication and Information Engineering, Shanghai University, Shanghai, 200072, China; 3 Key Laboratory of Systems Biology, SIBS-Novo Nordisk Translational Research Centre for PreDiabetes, Shanghai Institutes for Biological Sciences, Chinese Academy of Sciences, Shanghai, 200031, China; 4 LERIA, University of Angers, 2 Boulevard Lavoisier, 49045, Angers Cedex 01, France; 5 Institute of Industrial Science, University of Tokyo, Tokyo, 153-8505, Japan

## Abstract

**Background:**

Cancers, a group of multifactorial complex diseases, are generally caused by mutation of multiple genes or dysregulation of pathways. Identifying biomarkers that can characterize cancers would help to understand and diagnose cancers. Traditional computational methods that detect genes differentially expressed between cancer and normal samples fail to work due to small sample size and independent assumption among genes. On the other hand, genes work in concert to perform their functions. Therefore, it is expected that dysregulated pathways will serve as better biomarkers compared with single genes.

**Results:**

In this paper, we propose a novel approach to identify dysregulated pathways in cancer based on a pathway interaction network. Our contribution is three-fold. Firstly, we present a new method to construct pathway interaction network based on gene expression, protein-protein interactions and cellular pathways. Secondly, the identification of dysregulated pathways in cancer is treated as a feature selection problem, which is biologically reasonable and easy to interpret. Thirdly, the dysregulated pathways are identified as subnetworks from the pathway interaction networks, where the subnetworks characterize very well the functional dependency or crosstalk between pathways. The benchmarking results on several distinct cancer datasets demonstrate that our method can obtain more reliable and accurate results compared with existing state of the art methods. Further functional analysis and independent literature evidence also confirm that our identified potential pathogenic pathways are biologically reasonable, indicating the effectiveness of our method.

**Conclusions:**

Dysregulated pathways can serve as better biomarkers compared with single genes. In this work, by utilizing pathway interaction networks and gene expression data, we propose a novel approach that effectively identifies dysregulated pathways, which can not only be used as biomarkers to diagnose cancers but also serve as potential drug targets in the future.

## Background

Cancer is a type of complex diseases, which generally involves multiple gene mutations and pathway dysregulations [1,2]. Identifying biomarkers for cancer can help to understand and diagnose diseases, which in turn helps to design drugs with effective therapy. However, it is a challenging task to detect reliable biomarkers in cancers. Recently, the accumulation of large amount of “omics” data in public databases provides an opportunity for detecting biomarkers, among which the gene expression data are widely used. Accordingly, much effort has been made to identify causal disease genes based on these data. For example, many computational methods have been developed to detect differentially expressed genes between normal and disease samples [3-5], and these genes are supposed to be related to diseases and can be used as biomarkers. Unfortunately, many of the differentially expressed genes detected in one dataset are later found not to work effectively in another dataset for the same disease, especially for complex diseases [6]. This phenomenon may arise due to the independency assumption among disease related genes when detecting differentially expressed genes, whereas complex diseases are generally caused by the dysregulation of functional modules that consist of a set of genes [7-9].

Due to the poor performance of biomarkers as differentially expressed genes, some approaches have been proposed to identify possible pathogenic pathways, which improves the robustness and accuracy when these pathways are used as biomarkers compared with above mentioned gene based methods [10-18]. For example, Lee *et al.*[13] proposed to use a subset of genes belonging to one pathway as biomarkers to accurately distinguish diseases from controls. Liu *et al.*[18] used pathways to compare different regions of Alzheimer's disease brains and found dysfunctional pathways that cooperate in different brain regions. Despite the success of these methods on some datasets, the majority of them do not consider the functional dependency between pathways. Generally, different pathways have crosstalk with each other, and the deregulation of one pathway may affect the activities of many related pathways. Therefore, it is possible to detect more reliable pathway biomarkers by taking into account the functional dependency or interaction between pathways.

In this paper, we propose a novel method to identify dysregulated pathways by considering pathway interactions. The identified dysregulated pathways can be used as candidate biomarkers to diagnose cancer. Specifically, a new approach is proposed to construct a pathway interaction network, which describes the functional dependency between pathways. Subsequently, the dysregulated pathways in cancer are identified as the best features to discriminate cancers from controls in a machine learning framework. Benchmarking our method on several distinct cancer datasets shows that our method outperforms previous state of the art methods. Furthermore, functional analysis and independent experimental evidence demonstrate that our identified dysregulated pathways are biologically reasonable, indicating the practical efficiency of the proposed method.

## Methods

### Datasets

#### Gene expression data

The gene expression datasets were obtained from the NCBI Gene Expression Omnibus (GEO) [19]. We chose four different types of cancer datasets that have balanced number of disease and control samples in each dataset. Table 1 lists the gene expression datasets that were used in this work, including lung cancer (GSE4115) [20], prostate tumour (GSE6919) [21], breast cancer (GSE15852) [22], and pancreatic tumour (GSE16515) [23]. For each gene expression dataset, the annotations for probes were obtained from GEO and each probe was mapped to a gene, where the probes were discarded if they do not match any gene. The expression value averaged over probes was used as the gene expression value if the gene has multiple probes. Subsequently, the expression values of all genes in each dataset were standardized as follows

(1) $$z_{\mathrm{ij}}=\frac{g_{\mathrm{ij}}-mean(g_{i})}{std(g_{i})}$$

where *g*_*ij*_ represents the expression value of gene *i* in sample *j*, and *mean(g*_*i*_*)* and *std(g*_*i*_*)* respectively represents mean and standard deviation of the expression vector for gene *i* across all samples.

**Table 1:** Cancer gene expression datasets

**GEO accession number**	**Disease**	**Number of samples (Disease/Control)**	**Platform**
GSE 4115	Lung Cancer	187 (97/90)	GPL96 (HG-U133A)
GSE 6919	Prostate Tumour	128 (65/63)	GPL8300 (HG_U95Av2)
GSE 15852	Breast Tumour	86 (43/43)	GPL96 (HG-U133A)
GSE 16515	Pancreatic Tumour	52 (36/16)	GPL570 (HG-U133_Plus_2)

#### Cellular pathways and human protein-protein interactions

The predefined biological pathways were obtained from the Molecular Signatures Database (MSigDB) [24], which is a large collection of annotated functional gene sets. We chose the canonical pathways in the curated gene sets that contain 880 pathways, including the metabolic and signaling pathways collected from BioCarta (http://www.biocarta.com), KEGG [25], and Reactome [26]. The human protein-protein interactions (PPIs) were obtained from the Human Protein Reference Database (HPRD, downloaded in February 2010) [27], which contains manually curated protein-protein interactions. The PPI data set contains 38788 protein interactions among 9630 unique human proteins.

### Pathway activity and pathway interaction network

**Figure 1:**
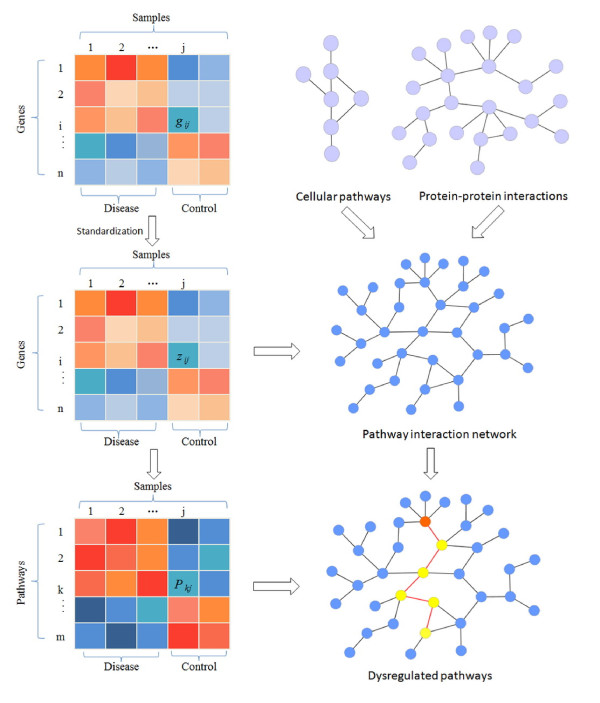
**Schematic illustration of identifying dysregulated pathway in cancer.** Firstly, gene expression profiles were standardized. Secondly, the genes were mapped to pathways. For each pathway, the principal component analysis (PCA) was employed to calculate the pathway activity score that summarizes the expression values of genes in each pathway. Thirdly, the pathway interaction network (PIN) was constructed based on gene expression data, protein-protein interactions, and cellular pathways. In the PIN, each node represents a pathway while each edge denotes the functional association between two pathways. Fourthly, the dysregulated pathways were identified as pathway markers that can best distinguish diseases from controls. The red node in PIN is the firstly identified pathway marker in disease, and the yellow ones are those pathway markers that can be combined with the first selected pathway to obtain best classification results while discriminating between diseases and controls.

Figure 1 illustrates the flowchart of our proposed method. Firstly, the pathway activity was defined based on gene expression data for each pathway. Secondly, a pathway interaction network (PIN) was constructed based on pathways and PPIs for each dataset. Thirdly, the dysregulated pathways in cancer are identified from PIN. The details were addressed as follows.

#### Pathway activity

All the genes were mapped to pathways extracted from MsigDB and only those genes that can be mapped to pathways were kept for further analysis hereinafter. After the genes were mapped to pathways, we defined an activity score for each pathway as the summary of the expression values of all genes belonging to this pathway. In particular, we used principal component analysis (PCA) method [28] to get the summary of all gene expressions of each pathway. The PCA technique can effectively characterize the internal structure of high-dimension dataset by preserving the variance in the data while transforming the data into low-dimension space. In brief, the activity score *P*_*kj*_ of pathway *k* in sample *j* was defined as follows.

(2) $$P_{\mathrm{kj}}=w_{1jk}z_{1jk}+w_{2jk}z_{2jk}\cdots +w_{\mathrm{ijk}}z_{\mathrm{ijk}}$$

where *z*_*ijk*_ represents the standardized expression value of gene *i* from pathway *k* in sample *j,* and *w*_*ijk*_ denotes weight for *z*_*ijk*_. In other words, the activity of each pathway can be regarded as the linear combination of the expressions of all genes in the pathway, and each pathway can be regarded as a meta-gene. In particular, the first principal component from PCA was used as the activity score for the corresponding pathway here. Therefore, the pathways that have different activities in diseases between controls are possibly related to diseases.

#### Pathway interaction network (PIN)

A pathway interaction network (PIN) was constructed with each node representing a pathway, where one edge was laid between two pathways if they share at least one gene or there are interactions between genes from the two pathways based on PPIs. Due to the condition-specificity of gene expression and pathway activity, for a given gene expression dataset, we further required that at least one of the common genes between two pathways is differentially expressed (student’s *t*-test, p-value < 0.05) between diseases and controls, or the two genes that code a pair of interacting proteins used to lay an edge between two pathways are highly co-expressed (Pearson’s correlation coefficient, absolute value > 0.8). Otherwise, the edge between two pathways will be removed. Therefore, a pathway interaction network was constructed for each dataset. The number of pathways and corresponding interactions in each PIN built for each dataset were shown in Table 2.

**Table 2:** The number of pathways and interactions for each dataset

**GEO accession number**	**Number of pathways**	**Number of interactions**	**Number of genes**
GSE 4115	867	43021	5371
GSE 6919	867	33123	4429
GSE 15852	866	40632	5325
GSE 16515	880	53397	6152

### Identifying dysregulated pathways from pathway interaction network

After defining the activity score for each pathway, we formulated the identification of dysregulated pathways as a feature selection problem in a machine learning framework, where the minimum set of pathways that can best discriminate diseases from controls were considered to be more possibly dysregulated pathways. It is reasonable and biologically interpretable to consider dysregulated pathways as discriminative features. In detail, a single pathway that can best discriminate between diseases and controls was firstly identified as the first pathway biomarker, and the second pathway that can be combined with the first pathway to get better classification results was identified from those pathways that interact with the first pathway in PIN. This procedure was repeated to add new pathways to selected pathway biomarkers until no more pathways can be added to improve classification accuracy, and the final selected pathway biomarkers were retained as potential dysregulated pathways in diseases. In feature selection, we used support vector machines (SVMs), which is a widely used kernel based method especially useful for small number of samples with high dimensional variables. In this work, the LIBSVM [29] toolbox was used with radial basis functional (RBF) kernel. The performance of the classifier was evaluated with five-fold cross validation, and AUC (Area Under ROC Curve) score was adopted as classification performance index. In the five-fold cross validation, all samples were randomly split into five equal-size subsets without overlap, four of which were used as training set while the rest one was used to evaluate the classification performance. To get robust results, we repeated five-fold cross-validation for 100 times and the average was used as the final result in each dataset.

## Results

### Identification of dysregulated pathways in cancer

**Figure 2:**
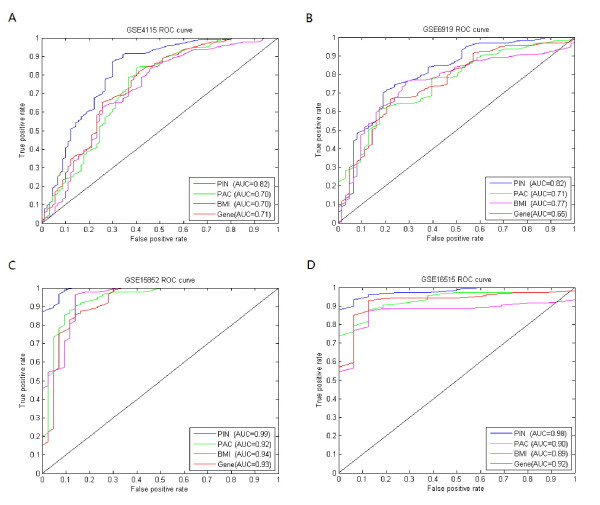
**Results obtained by PIN, PAC, BMI and gene biomarkers on four cancer datasets.** Results obtained by PIN, PAC, BMI and gene biomarkers on four cancer datasets, where PIN, PAC, BMI and Gene respectively denotes our pathway biomarkers, PAC biomarkers, BMI biomarkers and gene biomarkers. (**A**). Lung cancer dataset, where PIN gets AUC score of 0.82 compared with 0.70 by PAC, 0.76 by BMI and 0.73 by Gene. (**B**). Prostate tumour dataset, where PIN gets AUC score of 0.82 compared with 0.71 by PAC, 0.77 by BMI and 0.63 by Gene. (**C**). Breast tumour dataset, where PIN gets AUC score of 0.99 compared with 0.92 by PAC, 0.93 by BMI and 0.90 by Gene. (**D**). Pancreatic tumour dataset, where PIN gets AUC score of 0.98 compared with 0.90 by PAC, 0.84 by BMI and 0.90 by Gene.

To evaluate our method, we applied it to identify dysregulated pathways for the four cancer datasets listed in Table 1. Moreover, we used these pathways to discriminate diseases from controls and compared our results with two classical differentially expressed gene detection methods, including the student’s *t*-test and Biomarker identifier (BMI) method [30,31]. In the BMI method, the differentially expressed genes were ranked by logistic regression analysis (LRA), and this method was shown to outperform other methods. The genes selected by student’s *t*-test and BMI were respectively denoted as gene biomarkers and BMI biomarkers hereinafter. For comparison with student’s *t*-test and BMI, we picked the same number of top ranked genes by these two methods as that of our selected pathways. Figure 2 shows the results obtained by gene biomarkers and BMI biomarkers compared with our method (denoted as PIN biomarkers). The dysregulated pathways identified by our method in four cancer datasets were respectively listed in Tables 3,4,5,6. We can clearly see from the results that our method outperforms the other two methods on all four different cancer datasets, indicating the effectiveness and efficiency of our proposed method. For example, for lung cancer dataset, our method performed very well with an AUC score of 0.82 compared against gene biomarker with an AUC score of 0.71 and BMI biomarker with an AUC score of 0.70. Except for the AUC score, we also compared the four methods with respect to accuracy, sensitivity and specificity (detailed results can be found in Additional file 1: Table S1). The promising results obtained by the proposed method also demonstrate that our identified pathway biomarkers are potential dysregulated pathways in cancer.

**Table 3:** Dysregulated pathways identified in lung cancer (GSE4115) dataset

**Pathway**	**Description**	**Number of genes**
REACTOME_SPHINGOLIPID_METABOLISM	Genes involved in sphingolipid metabolism	32
REACTOME_TRIACYLGLYCERIDE_BIOSYNTHESIS	Genes involved in triacylglyceride biosynthesis	14
KEGG_PPAR_SIGNALING_PATHWAY	PPAR signaling pathway	69
REACTOME_AKT_PHOSPHORYLATES_TARGETS_IN_THE_CYTOSOL	Genes involved in AKT phosphorylates targets in the cytosol	14
REACTOME_TCR_SIGNALING	Genes involved in TCR signaling	64
BIOCARTA_STATHMIN_PATHWAY	Stathmin and breast cancer resistance to antimicrotubule agents	19
ST_T_CELL_SIGNAL_TRANSDUCTION	T Cell signal transduction	44
BIOCARTA_SPRY_PATHWAY	Sprouty regulation of tyrosine kinase signals	18
ST_IL_13_PATHWAY	Interleukin 13 (IL-13) Pathway	7
BIOCARTA_INTEGRIN_PATHWAY	Integrin signaling pathway	38
BIOCARTA_CELL2CELL_PATHWAY	Cell to cell adhesion signaling	14
REACTOME_APOPTOTIC_CLEAVAGE_OF_CELL_ADHESION_PROTEINS	Genes involved in apoptotic cleavage of cell adhesion proteins	11

**Table 4:** Dysregulated pathways identified in prostate tumour (GSE6919) dataset

**Pathway**	**Description**	**Number of genes**
REACTOME_METABLISM_OF_NUCLEOTIDES	Genes involved in metablism of nucleotides	71
REACTOME_PURINE_METABOLISM	Genes involved in purine metabolism	30
KEGG_NICOTINATE_AND_NICOTINAMIDE_METABOLISM	Nicotinate and nicotinamide metabolism	24
KEGG_TRYPTOPHAN_METABOLISM	Tryptophan metabolism	40

**Table 5:** Dysregulated pathways identified in breast tumour (GSE15852) dataset

**Pathway**	**Description**	**Number of genes**
KEGG_ADIPOCYTOKINE_SIGNALING_PATHWAY	Adipocytokine signaling pathway	67
REACTOME_TCR_SIGNALING	Genes involved in TCR signaling	64
REACTOME_P75NTR_SIGNALS_VIA_NFKB	Genes involved in p75NTR signals *via* NF-κB	13
BIOCARTA_ATM_PATHWAY	ATM signaling pathway	20
REACTOME_ACTIVATION_OF_THE_AP1_FAMILY_OF_TRANSCRIPTION_FACTORS	Genes involved in activation of the AP-1 family of transcription factors	10
KEGG_INSULIN_SIGNALING_PATHWAY	Insulin signaling pathway	137
BIOCARTA_AKAP13_PATHWAY	Rho-Selective guanine exchange factor AKAP13 mediates stress fiber formation	12
BIOCARTA_CK1_PATHWAY	Regulation of ck1/cdk5 by type 1 glutamate receptors	17
KEGG_PANCREATIC_CANCER	Pancreatic cancer	70

**Table 6:** Dysregulated pathways identified in pancreatic tumour (GSE16515) dataset

**Pathway**	**Description**	**Number of genes**
KEGG_P53_SIGNALING_PATHWAY	P53 signaling pathway	69
ST_JNK_MAPK_PATHWAY	JNK MAPK pathway	38
ST_P38_MAPK_PATHWAY	P38 MAPK pathway	35
BIOCARTA_SALMONELLA_PATHWAY	Salmonella pathway	13
BIOCARTA_CDC42RAC_PATHWAY	Role of PI3K subunit p85 in regulation of actin organization and cell migration	16

Moreover, we also compared our method with one state of the art dysregulation pathway identification method, *i.e.*, PAC (Pathway Activity inference using Condition-responsive gene activity) method, proposed by Lee et al. [13]. In the PAC method, the pathway activity was defined as a combined score of a subset of genes, called the condition-responsive genes, that yields the best discriminative score. The pathways with different discriminative power were subsequently ranked based on *t*-test. We performed the PAC method on above four cancer datasets. For a fair comparison, we used the same SVM toolbox and the same number of pathways identified by our method. The results of the PAC method (denoted as PAC biomarkers) were also shown in Figure 2 (detailed results can be found in Additional file 1: Table S1). As shown in Figure 2, our proposed method achieved a higher AUC score than the PAC method on all four datasets. These results indicate that our proposed approach helps to improve the discriminative power by taking into account the functional dependency between pathways.

Furthermore, we compared the genes involved in our identified dysregulated pathways with those top ranked differentially expressed genes. Table 7 lists the numbers of genes involved in both our identified dysregulated pathways and those top ranked differentially expressed genes (the same number of genes as those in dysregulated pathways). It is found that only a small fraction (from 2.8% to 8.4%) of the genes in our identified dysregulated pathways overlaps with top ranked differentially expressed genes. This phenomenon implies that a pathway as an entity can better diagnose complex diseases rather than individual genes even though the genes in the pathway are not differentially expressed significantly.

**Table 7:** The overlap between the genes in dysregulated pathways and gene biomarkers, where the two sets have the same number of genes

**GEO accession number**	**Number of genes in pathway biomarkers**	**Overlap with top ranked gene biomarkers**	**Percentage**
GSE 4115	255	9	3.5%
GSE 8397	94	5	5.3%
GSE 15852	285	24	8.4%
GSE 16515	142	4	2.8%

### Dysregulated pathways are robust as biomarkers

**Figure 3:**
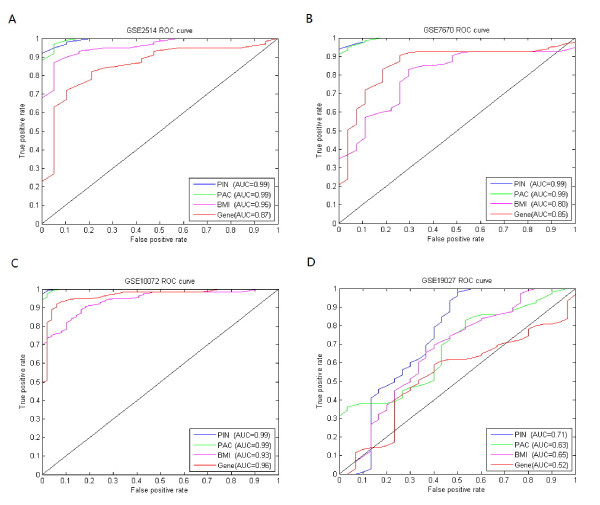
**Results obtained by PIN, PAC, BMI and gene biomarkers on four lung cancer datasets.** The biomarkers identified from lung cancer dataset (GSE 4115) by four methods were applied to independent lung cancer test datasets (GSE7670, GSE10072, GSE19027, and GSE2514), where PIN, PAC, BMI and Gene respectively denotes our pathway biomarkers, PAC biomarkers, BMI biomarkers and gene biomarkers. (**A**). GSE2514 dataset, where PIN gets AUC score of 0.99 compared with 0.99 by PAC, 0.95 by BMI and 0.87 by Gene. (**B**). GSE7670 dataset, where PIN gets AUC score of 0.99 compared with 0.99 by PAC, 0.80 by BMI and 0.85 by Gene. (**C**). GSE10072 dataset, where PIN gets AUC score of 0.99 compared with 0.99 by PAC, 0.93 by BMI and 0.96 by Gene. (**D**). GSE19027 dataset, where PIN gets AUC score of 0.71 compared with 0.63 by PAC, 0.65 by BMI and 0.52 by Gene.

To further test our method, we applied the identified dysregulated pathways from above lung cancer dataset (GSE 4115, see Table 3) to other four independent hold-out datasets of lung cancer (GSE2514 [32], GSE7670 [33], GSE10072 [34] and GSE19027 [35]) that are from two different Affymetrix platforms, *i.e.*, GPL8300 (HG_U95Av2) and GPL96 (HG-U133A). Note that all of these test datasets list in Table 8 are not used in above section, thereby evaluating our proposed method in an objective way. Similarly, the gene or pathway biomarkers selected from GSE4115 dataset by other methods were also applied to the four lung cancer test datasets. The same numbers of pathways or genes as that of our selected pathways were chosen for a fair comparison. The pathway biomarkers identified by our method achieved higher or comparable AUC scores compared with other methods on all four datasets. For example, in GSE19027 dataset, our pathway biomarkers got an AUC score of 0.71 compared with 0.63 by PAC biomarkers, 0.65 by BMI biomarkers and 0.52 by gene biomarkers. Figure 3 shows the results obtained with biomarkers identified by our method compared with the other three methods. Furthermore, we also compared the four methods with respect to accuracy, sensitivity and specificity. For the dataset of GSE10072, both PIN biomarkers and PAC biomarkers achieved an AUC score of 0.99 compared with 0.93 by BMI biomarkers and 0.96 by gene biomarkers. However, PIN biomarkers achieved the highest sensitivity and specificity. The detailed results can be found in Additional file 2: Table S2. The good performance of our method on both training dataset and the four independent test dataset demonstrates that our identified dysregulated pathways can serve as robust biomarkers.

**Table 8:** The four lung cancer test datasets

**GEO accession number**	**Number of samples (Disease/Control)**	**Platform**
GSE 2514	39 (19/20)	GPL8300 (HG_U95Av2)
GSE 7670	54 (27/27)	GPL96 (HG-U133A)
GSE 10072	107 (49/57)	GPL96 (HG-U133A)
GSE 19027	51 (30/21)	GPL96 (HG-U133A)

### Dysregulated pathways provide insights into pathogenesis of cancer

**Figure 4:**
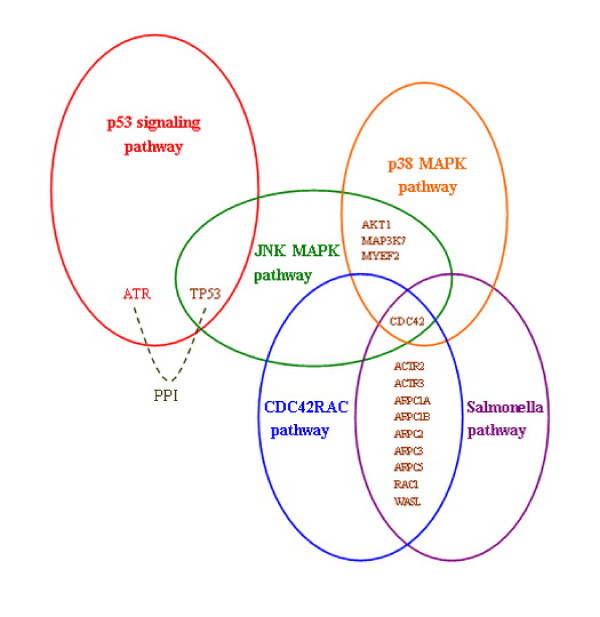
**Dysregulated pathways interaction network in pancreatic tumour dataset.** In pancreatic tumour dataset (GSE16515), five dysregulated pathways were identified which can be assembled into a network based on their interactions in the pathway interaction network constructed for this dataset. Different colours were used to represent the five dysregulated pathways. The common genes between pathways are differentially expressed and the dashed line between two genes from distinct dysrugulated pathways denotes protein-protein interaction.

We further investigated the five identified dysregulated pathways in pancreatic cancer (see Table 6). From the pathway list, we can find that some identified dysregulated pathways involve hallmark cancer genes, such as P53, NF-κB, PI3K, *etc.* Figure 4 shows the interactions among the five identified dysregulated pathways in PIN, including P53 signaling pathway, JNK MAPK pathway, P38 MAPK pathway, Salmonella pathway, and CDC42RAC pathway, where the last four pathways connect with each other.

P53 is a well-known tumour suppressor gene, which is involved in various biological processes, including cell cycle, apoptosis and senescence, *etc.*[36]. Mutations that deactivate P53 were found in most tumour types, and P53 plays an important regulation role in tumour progression. Interestingly, P53 signaling pathway was identified as the top dysregulated pathway by our method. The JNK MAPK pathway interacts with P53 signaling pathway. Jun N-terminal kinase (JNK) is one of mitogen-activated protein kinase (MAPK) members and also a stress-activated protein kinase. Both P53 and JNK are two important apoptosis-regulatory factors frequently deregulated in cancer cells. They also participate in the modulation of autophagy and can be regulated by TNF alpha (tumour necrosis factor alpha), which is a soluble cytokine mediator of immune responses and involved in various biological functions. JNK and ERK mediate TNF alpha-induced P53 activation in apoptosis and autophagic activity. Another identified desregulated pathway P38 MAPK pathway is also involved in this process, where P38 is one member of the MAPK superfamily. JNK and P38 MAPK pathways that are activated by stress and inflammatory signals have crosstalk, thereby working together to affect proliferation, differentiation, survival, and migration. The P38 MAPK pathway can negatively regulate JNK activity in several contexts [37]. TNF alpha regulates the JNK and P38 MAPKs in apoptotic and autophagic process in which ERK/JNK plays a promoting role while P38 plays an inhibiting one [38]. JNK activation can also be negatively regulated by NF-κB which is widely involved in oncogenesis, cell proliferation and apoptosis, and evasion of immune responses [39]. Inhibition of NF-κB activation and sustained JNK activation promote the TNF alpha mediated cell apoptotic and suppress the tumour progression [40]. The Salmonella pathway and CDC42RAC pathway are both related to cell invasion and migration. Cdc42 gene is the common differentially expressed gene in four dysregulated pathways indicating its key role in pancreatic tumour. The CDC42RAC pathway regulates cell migration through P85 that is a subunit of PI3Ks (Phosphatidylinositol-3 kinases). P85 activates Cdc42 which affects the formation of new actin fibers and interacts with Wiskott–Aldrich syndrome protein (WASP) to stimulate migration [41]. On the other hand, activated P85 can bind to P110, another subunit of PI3K, which can activate Akt through PIP3 that serves as a second messenger. Akt plays a main role in cell survival, proliferation, and growth [42]. The mutation of P85 and activation of Akt have been found in some primary tumours, including the pancreatic tumour [43].

Furthermore, we applied NOA (Network Ontology Analysis) web tools [44] to identify enriched GO function for genes in our identified dysregulated pathways. The top 5 enriched GO biological processes for each pathway biomarker in pancreatic tumour dataset were listed in Table 9. From the analysis, we found that those enriched processes, such as regulation of cell cycle, apoptosis and regulation of cellular component biogenesis, are most important biological processes in tumour progression, thereby implying the effectiveness of our proposed method. The identified enriched GO terms on the other three cancer datasets were listed in Additional file 3: Table S3.

**Table 9:** The top 5 enriched GO terms for each dysregulated pathway in pancreatic tumour (GSE16515) dataset

**Pathway**	**GO: term**	**p-value**	**Term name**
KEGG_P53_SIGNALING_PATHWAY	GO:0051726	1.30E-33	regulation of cell cycle
	GO:0006917	1.30E-22	induction of apoptosis
	GO:0012502	1.50E-22	induction of programmed cell death
	GO:0006915	1.10E-20	apoptosis
	GO:0042981	1.20E-20	regulation of apoptosis
ST_JNK_MAPK_PATHWAY	GO:0000165	6.70E-33	MAPKKK cascade
	GO:0023014	8.90E-27	signal transmission *via* phosphorylation event
	GO:0007243	8.90E-27	intracellular protein kinase cascade
	GO:0031098	1.40E-23	stress-activated protein kinase signaling cascade
	GO:0007254	8.60E-22	JNK cascade
ST_P38_MAPK_PATHWAY	GO:0044087	1.40E-13	regulation of cellular component biogenesis
	GO:0043254	4.70E-13	regulation of protein complex assembly
	GO:0030833	3.30E-12	regulation of actin filament polymerization
	GO:0030036	4.40E-12	actin cytoskeleton organization
	GO:0008064	6.40E-12	regulation of actin polymerization or depolymerization
BIOCARTA_SALMONELLA_PATHWAY	GO:0006793	1.70E-17	phosphorus metabolic process
	GO:0006796	1.70E-17	phosphate metabolic process
	GO:0006468	4.10E-17	protein amino acid phosphorylation
	GO:0016310	1.30E-16	phosphorylation
	GO:0043687	9.20E-16	post-translational protein modification
BIOCARTA_CDC42RAC_PATHWAY	GO:0044087	1.20E-14	regulation of cellular component biogenesis
	GO:0043254	3.10E-12	regulation of protein complex assembly
	GO:0032956	3.30E-12	regulation of actin cytoskeleton organization
	GO:0032970	4.20E-12	regulation of actin filament-based process
	GO:0030833	1.50E-11	regulation of actin filament polymerization

## Discussion

Identifying biomarkers in complex diseases can help diagnose disease and design more effective drugs. The accumulation of “omics” data, especially gene expression data, makes it possible to detect biomarkers in a more efficient way [45,46]. However, it is a challenging task to identify robust biomarkers from about 20,000 genes considering that complex diseases are usually caused from mutations of multiple correlated genes or failure of certain subsystems rather than individual genes. Traditional methods detecting differentially expressed genes as biomarkers failed to work in some cases due to the independent assumption among genes, whereas complex diseases generally affect a set of functionally related genes.

In this paper, we proposed a novel method to identify dysregulated pathways in cancer. Unlike the existing methods, our method considers the functional dependency between pathways by constructing a pathway interaction network. Benchmarking our method on several different cancer datasets demonstrates the effectiveness of the proposed method. The results on independent test datasets imply the robustness of our identified pathway biomarkers. Further analyses indicate that the dysregulated pathways that we identified are indeed involved in tumour processes, and some of these dysregulated pathways may serve as drug targets in the near future [37]. Therefore, the functional relationship between pathways can not only provide insights into disease mechanisms but also provide alternative ways to develop more efficient drugs.

## Conclusions

In this work, we present a novel approach to identify dysregulated pathways in cancer based on a derived pathway interaction network that describes the functional dependency between pathways. The promising results obtained by our method indicate that the dysregulated pathways indeed have crosstalk with each other. The comparison between our method and other state of the art methods on multiple cancer datasets demonstrates that our identified dysregulated pathways can serve as robust biomarkers. We believe that our proposed method can help to predict new biomarkers and even drug targets in a more accurate and robust way.

## Authors' contributions

XMZ conceived the idea and designed the experiments. KQL implemented the algorithm. KQL, XMZ, LNC, JKH, and ZPL wrote the manuscript. All authors read and approved the final manuscript.

## Supplementary Material

Additional file 1 Table S1 **Classification results on four cancer datasets based on the identified dysregulated pathways.** The classification results on four distinct cancer (lung cancer, prostate tumour, breast tumour and pancreatic tumour) datasets by pathway biomarkers, compared with PAC biomarkers, BMI biomarkers and gene biomarkers. (DOC 49 kb) Click here for file

Additional file 2 Table S2 **The results of our identified dysregulated pathways on lung cancer test dataset.** The results of our identified dysregulated pathways on lung cancer test dataset, compared with PAC biomarkers, BMI biomarkers and gene biomarkers. (DOC 53 kb) Click here for file

Additional file 3 Table S3 **The list of identified enriched GO term in dysregulated pathway biomarkers.** The list of top 5 enriched GO biological processes of pathway biomarkers in lung cancer (GSE4115) dataset, prostate tumour (GSE6919) and breast tumour (GSE15852) dataset. (XLS 31 kb) Click here for file
