# Supplementary material for: Identifying dysregulated pathways in cancers from pathway interaction networks
Source: BMC Bioinformatics. 2012 Jun 7;13:126. doi: 10.1186/1471-2105-13-126 (PMC3443452; doi:10.1186/1471-2105-13-126)
Supplement: Additional file 1 — Table S1 Classification results on four cancer datasets based on the identified dysregulated pathways. The classification results on four distinct cancer (lung cancer, prostate tumour, breast tumour and pancreatic tumour) datasets by pathway biomarkers, compared with PAC biomarkers, BMI biomarkers and gene biomarkers. (DOC 49 kb) [file 1471-2105-13-126-S1.doc]

**Classification results on four cancer datasets based on the identified dysregulated pathways**

The classification results on four distinct cancer (lung cancer, prostate tumour, breast tumour and pancreatic tumour) datasets by pathway biomarkers, compared with PAC biomarkers, BMI biomarkers and gene biomarkers.

| GEO accession number | Index | Pathway biomarkers | PAC biomarkers | BMI biomarkers | Gene biomarkers |
| --- | --- | --- | --- | --- | --- |
| GSE4115 | AUC | **0.82** | 0.70 | 0.70 | 0.71 |
|  | sensitivity | **0.81** | 0.74 | 0.74 | 0.66 |
|  | specificity | **0.70** | 0.58 | 0.58 | 0.64 |
|  | accuracy | **0.76** | 0.66 | 0.66 | 0.65 |
| GSE6919 | AUC | **0.82** | 0.71 | 0.77 | 0.65 |
|  | sensitivity | **0.73** | 0.64 | 0.66 | 0.56 |
|  | specificity | 0.74 | 0.67 | **0.76** | 0.70 |
|  | accuracy | **0.73** | 0.65 | 0.71 | 0.63 |
| GSE15852 | AUC | **0.99** | 0.92 | 0.94 | 0.93 |
|  | sensitivity | **0.96** | 0.88 | 0.84 | 0.86 |
|  | specificity | **0.93** | 0.83 | 0.87 | 0.85 |
|  | accuracy | **0.94** | 0.85 | 0.85 | 0.86 |
| GSE16515 | AUC | **0.98** | 0.90 | 0.89 | 0.92 |
|  | sensitivity | **0.96** | 0.91 | 0.93 | 0.83 |
|  | specificity | 0.82 | 0.67 | 0.74 | **0.87** |
|  | accuracy | **0.92** | 0.84 | 0.72 | 0.84 |
