# Supplementary material for: Identifying dysregulated pathways in cancers from pathway interaction networks
Source: BMC Bioinformatics. 2012 Jun 7;13:126. doi: 10.1186/1471-2105-13-126 (PMC3443452; doi:10.1186/1471-2105-13-126)
Supplement: Additional file 2 — Table S2 The results of our identified dysregulated pathways on lung cancer test dataset. The results of our identified dysregulated pathways on lung cancer test dataset, compared with PAC biomarkers, BMI biomarkers and gene biomarkers. (DOC 53 kb) [file 1471-2105-13-126-S2.doc]

**The results of our identified dysregulated pathaways on lung cancer test dataset**

The results of our identified dysregulated pathaways on lung cancer test dataset, compared with PAC biomarkers, BMI biomarkers and gene biomarkers.

| GEO accession number | Platform | Index | PIN biomarkers | PAC biomarkers | BMI biomarkers | Gene biomarkers |
| --- | --- | --- | --- | --- | --- | --- |
| GSE2514 | GPL8300 (HG_U95Av2) | AUC | **0.99** | **0.99** | 0.95 | 0.87 |
|  |  | sensitivity | 0.95 | **0.97** | 0.85 | 0.75 |
|  |  | specificity | **0.99** | **0.99** | 0.91 | 0.84 |
|  |  | accuracy | 0.97 | **0.98** | 0.88 | 0.78 |
| GSE7670 | GPL96 (HG-U133A) | AUC | **0.99** | **0.99** | 0.80 | 0.85 |
|  |  | sensitivity | **0.94** | **0.94** | 0.71 | 0.75 |
|  |  | specificity | 0.96 | **0.97** | 0.74 | 0.82 |
|  |  | accuracy | **0.95** | **0.95** | 0.73 | 0.78 |
| GSE10072 | GPL96 (HG-U133A) | AUC | **0.99** | **0.99** | 0.93 | 0.96 |
|  |  | sensitivity | **0.99** | 0.98 | 0.85 | 0.89 |
|  |  | specificity | **0.98** | 0.96 | 0.83 | 0.91 |
|  |  | accuracy | **0.99** | 0.97 | 0.84 | 0.90 |
| GSE19027 | GPL96 (HG-U133A) | AUC | **0.71** | 0.63 | 0.65 | 0.52 |
|  |  | sensitivity | 0.43 | **0.45** | 0.27 | 0.15 |
|  |  | specificity | 0.76 | 0.71 | 0.82 | **0.86** |
|  |  | accuracy | **0.61** | 0.6 | 0.59 | 0.57 |
